# A Review of the Ongoing Research on Zika Virus Treatment

**DOI:** 10.3390/v10050255

**Published:** 2018-05-14

**Authors:** Suely da Silva, Daniel Oliveira Silva Martins, Ana Carolina Gomes Jardim

**Affiliations:** 1 Laboratory of Virology, Institute of Biomedical Science, ICBIM, Federal University of Uberlândia, Uberlândia, MG 38405-302, Brazil; suelysilva0712@hotmail.com (S.d.S.); danielosmartins@gmail.com (D.O.S.M.); 2 Genomics Study Laboratory, São Paulo State University, IBILCE, São José do Rio Preto, SP 15054-000, Brazil

**Keywords:** Zika fever, ZIKV, treatment, antiviral, screen of drugs

## Abstract

The Zika fever is an arboviral disease resulting from the infection with Zika virus (ZIKV). The virus is transmitted to humans by the bite of *Aedes* mosquitos, mainly *Aedes aegypti* and *Aedes albopictus*. ZIKV has been detected for decades in African and Asian regions and, since 2007, has spread to other continents; among them, infections are most reported in the Americas. This can be explained by the presence of vectors in highly populated and tropical regions where people are susceptible to contamination. ZIKV has been considered by the World Health Organization a serious public health problem because of the increasing number of cases of congenital malformation and neurological disorders related to its infection, such as microcephaly, Guillain–Barré syndrome, meningoencephalitis, and myelitis. There is no vaccine or specific antiviral against ZIKV. The infection is best prevented by avoiding mosquito bite, and the treatment of infected patients is palliative. In this context, the search for efficient antivirals is necessary but remains challenging. Here, we aim to review the molecules that have been described to interfere with ZIKV life cycle and discuss their potential use in ZIKV therapy.

## 1. Introduction

Zika virus (ZIKV) (strain MR 766) was first isolated [1] from the serum sample of a Rhesus monkey during a research on yellow fever virus (YFV) in the Zika forest, Uganda 1947. In 1948, the virus was isolated from a pool of *Aedes Africanus* (*Stegomya*) mosquitoes in the same forest [2,3,4]. The first cases of human infection were reported in 1950 in Africa and later in Asia and remained restricted to these regions until 2007 [2,5,6]. Since then, ZIKV has spread geographically, with reports of infection in the North Pacific [7,8,9], French Polynesia, and South Pacific [10]. The first records in the Americas date from 2015, when ZIKV spread across the Pacific Ocean to invade Brazil, Suriname, and Columbia [11]. Since 2015, 84 countries reported the new introduction or re-introduction of ZIKV, and, although prevention and control measures are being taken, the risk of infection is still a reality, mainly in regions with a large mosquito vector presence [12].

ZIKV is mainly transmitted by the bite of female mosquitoes of the *Aedes aegypti* and *Aedes albopictus* species. The latter is prevalent in Southeast Asia and, because of its ability to adapt to different environments, it has has been found in the Americas, Pacific Islands, Australia, and Africa. *A. albopictus* is a potential vector for more than 20 arboviruses, including important members of the Flaviviridae and Togaviridae families such as Dengue (DENV) and Chikungunya (CHIKV), respectively [13]. Both species can co-exist in the same regions. Although previous researches demonstrated the superiority of *A. albopictus*, this specie is considered the second main vector of DENV and CHIKV [13,14]. Indeed, the transmission by other species of the *Aedes* genus, including *Aedes africanus, Aedes luteocephalus, Aedes vitattus, Aedes furcifer, Aedes hensilii*, and *Aedes apicoargenteus*, should be considered [15]. The successful virus transmission by these species is related to their high capacity of proliferation in tropical and subtropical regions [15,16,17]. Even though wild primates are the main non-human reservoirs, anti-ZIKV antibodies have been also identified in rodents and domestic animals [18,19]. Additionally, others transmission routes have been confirmed, such as blood transfusion [20], sexual [21], perinatal, and transplacental transmissions [11,22].

Zika fever is considered a mild disease with symptoms as headache, fever, malaise, and joint pain [23]. However, since reemerged, ZIKV has been associated with the increasing number of microcephaly cases, Guillain–Barré syndrome, meningoencephalitis, and myelitis in highly affected areas like the Brazilian North Eastern State [24,25].

## 2. The ZIKA Virus

ZIKV belongs to the Flaviviridae family and the genus *Flavivirus*, being phylogenetically related to the dengue virus (DENV), West Nile virus (WNV), and yellow fever virus (YFV) [7]. Recently, the structural similarity among the virions of ZIKV and other *Flavivirus* was demonstrated by cryoelectron microscopy (cryo-EM) [26]. The viral nucleocapsid of *Flavivirus* is surrounded by a bilayer lipid membrane with a 25 to 30 nm diameter derived from the host cells, in which viral glycoproteins are inserted.

**Figure 1:**
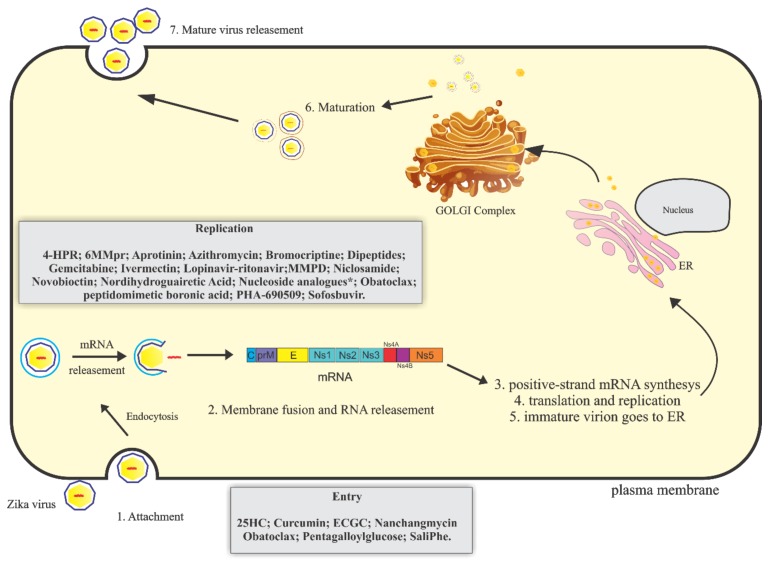
Schematic representation of Zika virus (ZIKV) replicative cycle. ZIKV replicative cycle and inhibitory drugs of viral entry and replication. 4-HPR = N(4-hydroxiphenyl) retinamide; 6MMpr = 6-methylmercaptopurine riboside; MMPD = Merimepodib; 25HC = 25-Hydroxycholesterol ; ECGC = Epigallocatechin galato; ***7-desaza-2′-C-methyladenosine (7-desaza-2′-CMA); 2′-C-methyladenosine (2′-CMA); 2′-C-methylcytidine (2′ CMC); 2′-C-methylguanosine (2′-CMG) and 2′-C-methyluridine (2′-CMU).

Like others flaviviruses, the virus enters the cell by endocytosis driven by the interaction of the envelope proteins and specific receptors on the host cell [27]. The process of internalization is intermediated by clathrin protein in a low pH environment. The viral genome is released into the host cytoplasm where it will be translated. The ZIKV genome is a positive-sense single-stranded RNA approximately 11 kb long that is uncoated immediately after virus entry into the host cells [28]. The viral genome has a single “open reading frame” (ORF) flanked by 5′ and 3′ untranslatable regions of 106 nucleotides and 428 nucleotides, respectively. The polyprotein encoded by this ORF is proteolytically cleaved into three structural proteins (capsid protein, premembrane/membrane protein, and envelope glycoprotein) and seven non-structural proteins (NS1, NS2A, NS2B, NS3, NS4A, NS4B, and NS5) [8,29,30]. The NS3 and NS5 proteins are essential for viral replication, possessing high catalytic activity [31]. After viral genome translation, the new viral particles are released from the cells and restart the infection process [30] (Figure 1).

Phylogenetic analysis of the ZIKV genome indicates the existence of three lineages: West Africa, Asian/American [32,33], and Brazilian ZIKV [34]. Further analyses of Asiatic and African ZIKV strains isolated from infected mosquitoes, monkeys, and humans showed significant amino acid variations throughout the sequence of the viral polyprotein [35]. The same study associated the human strains isolated during the recent outbreaks with the viral strain P6-740 (from Asian mosquito, 1966) and demonstrated that all strains have a common ancestor. However, all the recent strains present a minimum of 400 amino acid mutations when compared with P6-740, which can interfere with the viral replicative efficiency, fitness, and transmissibility [35].

## 3. Therapeutical Potential of Anti-Zikv Molecules

Currently, there is no approved vaccine or specific antiviral against ZIKV infection. The main strategy for controlling ZIKV is to prevent the breeding of mosquitoes [36]. The treatment is palliative and includes rest and fluids intake. Acetaminophen or paracetamol are used to alleviate the symptoms of headache, fever, and myalgia. The use of aspirin or other salicylates is not recommended in childhood to avoid the risk of Reye′s syndrome. Other non-steroidal anti-inflammatory drugs (NSAIDS) should be avoided due to the risk of hemorrhagic complications [37,38]. Here, we aim to review the molecules that have been described to interfere with the ZIKV replication life cycle (Figure 1, Table 1).

### 3.1. Drug Repurposing

A research group screened 6000 pharmacologically active compounds against ZIKV infection, measuring caspase-3 activity and cell viability of treated human neural cells. Emricasan, also known as IDN-6556 or PF-03491390, is an inhibitor of the pan-caspase pathway that presented a protective effect against ZIKV-induced cell death in gliobastoma SNB-19 cells. This effect was observed when the cells were previously infected with ZIKV strains MR766 (1947 Ugandan strain), FSS13025 (2010 Cambodian strain), PRVAB659 (2015 Puerto Rican strain) and then treated with 0.13–0.9 µM (IC50) of the compound. Also, Emricasan reduced the number of active (cleaved) caspase-3-expressing forebrain-specific human neural progenitor cells (hNPCs) infected by the FSS13025 strain in both monolayer and 3D organoid cultures. Although cell proliferation was not affected by Emricasan treatment of ZIKV-exposed brain organoids, the data showed that ZIKV antigen persisted in both cultures. Therefore, the authors concluded that Emricasan presented neuroprotective activity for hNPCs but did not inhibited ZIKV-replication [40].

Niclosamide, an anti-helminthic approved by the Food Drug Administration (FDA), and the cyclin-dependent kinase inhibitor PHA-690509 demonstrated to significantly inhibit ZIKV infection in vitro [40]. SNB-19 cells were treated with increasing concentrations of these compounds one hour prior inoculation with the FSS-13025, PRVABC59, or MR766 ZIKV strains (multiplicity of infection (MOI) = 1). Twenty-four hours post inoculation, the cells were harvested, and the expression of the protein NS1 was evaluated by western blotting. The results demonstrated that these compounds significantly inhibited infection by the three strains of ZIKV. Additionally, the effects of these compounds on the early and post-entry stages were investigated. The results showed that niclosamide and PHA-690509 inhibited ZIKV infection post-entry, probably blocking viral replication. Additionally, a combined therapy with Emricasan and PHA-690509 presented an additional inhibition effect on caspase activity, showing the synergistic effect of these compounds [40].

Similar analyses were performed by Barrows and coworkers who investigated the anti-ZIKV activity of a library of 774 drugs approved by the Food and Drug Administration (FDA). Huh-7.0 cells were pre-treated with the compounds for one hour, inoculated with ZIKV MEX_I_7/15 (MOI = 0.4), fixed twenty-four hours post-infection (h.p.i), and labeled with immunofluorescent antibodies against the viral envelope proteins. The majority of drugs showed no significant effect; however, 30 compounds demonstrated to be effective, decreasing virus infectivity. Eight compounds (ivermectin, daptomycin, mycophenolic acid (MPA), sertraline, pyrimethamine, cyclosporine A, azathioprine, and mefloquine) were selected for tests in human cell lines derived from cervical cancer (HeLa), placental (JEG3), and neural tissues (hNSC). In Hela cells, all the eight compounds reduced the infectivity rates, and four of these compounds presented anti-ZIKV activity in JEG3 cells. For flow cytometry analyses, hNSC cells were maintained in the presence of the drugs and ZIKV (MOI = 3) for 96 h. The results showed that MPA, cyclosporine A, and ivermectin significantly inhibited ZIKV MEX_I_7 infection. In human amnion epithelial cells (HAECs), ivermectin, daptomycin, sertraline, and mefloquine inhibited viral infection at the maximum concentration of 16 μM. Ivermectin, sertraline, and mefloquine demonstrated a higher antiviral effect, however, a significant reduction of cell viability was observed. Ivermectin (EC50 = 1 µM) and MPA (EC50 = 10 µM) demonstrated the most potent action on ZIKV replication [41]. Among these compounds, the most potent inhibitors were ivermectin, mycophenolic acid (MPA), and daptomycin. Ivermectin is an anti-helminthic agent that in previous studies showed a potential activity against in YFV infection [61], while MPA is a nonnucleoside inhibitor of inosine monophosphate (IMP) dehydrogenase whose antiviral properties against DENV have been described [62]. Daptomycin is an antimicrobial whose antiviral properties had not been previously described [41].

The antiviral activity of 2177 compounds approved by the FDA was evaluated as a treatment of ZIVK infection in vitro. The Vero and U87 cell lines were treated for a period of 2 h with each compound at 2 μM and then were infected with the strain ZIKV-BR, with MOI = 1, 3, or 10. After 72 h, the antiviral activity was analyzed by luminescence, and, in parallel, cell viability was evaluated. The compounds that inhibited viral infection and resulted in cell viability being 2.5-fold greater than in the untreated control were chosen to be validated by flow cytometry. To validate the antiviral properties, U87 cells were pretreated with different concentrations of the compounds for 1 h and then were infected with the Brazilian viral strains ZIKV-BR or ZIKV-PR. Forty-eight hours post-infection (h.p.i), the cells were fixed, permeabilized, labeled with specific antibodies against the viral envelope protein, and analyzed by flow cytometry. The macrolide antibiotic azithromycin inhibited viral replication at the different MOIs = 0.01 (EC50 = 2.1), MOI = 0.1 (EC50 = 2.9), and MOI = 3 (EC50 = 5.1) and also reduced cell death triggered by the infection. The authors suggested that this compound may be considered as a potential antiviral to treat the viral infection caused by ZIKV and could be administered to pregnant women [63].

Chloroquine is another compound approved by the FDA to treat malaria that can be administrated to pregnant women. The anti-ZIKV properties of chloroquine were tested in Vero cells, human brain microvascular endothelial cells (hBMEC), and hNSCs infected with ZIKV MR766 for 5 days and treated with the compound at concentrations ranging from 6.25 to 50 µM. The viral envelope E protein was labeled with the 4G2 antibody, specific to *flavivirus*, to measure viral infectivity. The data obtained showed a decreasing virus infection in a dose-dependent manner. The results were corroborated by immunofluorescence and flow cytometry analyses, which demonstrated that chloroquine at 50 µM reduced ZIKV infection up to 65% and 50% in Vero cells and hBMEC, respectively, with no effect on cell viability. In hNSCs, chloroquine at 50 µM reduced ZIKV infection by 57% after 4 days of treatment, with 70% of cell viability. Chloroquine at 25 µM was also tested in Vero cells infected with the ZIKV Brazilian strain isolate and showed to decrease up to 16 folds the viral RNA levels in the supernatant. Moreover, chloroquine activity was tested in distinct stages of ZIKV infection, demonstrating to block the early stages of the replication cycle [42]. Chufeng Li and coworkers also demonstrated that chloroquine protects fetal mice from microcephaly caused by ZIKV [64].

Bromocriptine is a dopamine D2 and D3 receptor agonist, which is used to treat galactorrhea and Parkinson′s disease [65,66] and was also reported to possess anti-viral properties [67]. Bromocriptine was tested for the ability to inhibit viral infection caused by the ZIKV. First, Vero cells were treated with different concentrations of the compound for 2 h. Then, the medium-containing compound was removed, and the cells were infected with ZIKV (Puerto Rico-PRVABC59) at MOI = 0.0001 for 1 h. After the adsorption, the inoculum was completely removed, and the cells were supplied with fresh media containing Bromocriptine and incubated for 6 days. The cytopathic effect was examined by microscopy, and cell viability was measured by the 3-(4,5-Dimethylthiazol-2-*yl*)-2,5-Diphenyltetrazolium Bromide (MTT) assay. The data showed the absence of cytopathic effects caused by ZIKV infection. In addition, the supernatant from the infected cells was collected for analysis of viral yield by qRT-PCR, which demonstrated a reduction of the viral titers, suggesting that Bromocriptine is able to inhibit the infection caused by ZIKV. In order to evaluate in which stage of the replicative cycle the compound was acting, a time-of-drug addiction assay was performed. The cells were treated with 20 µM of bromocriptine prior to the infection (−1 to 0 h.p.i), simultaneously with the virus (0 to 1 h.p.i), and after infection (0.3, 6, 9, 12, 14 h.p.i). The cells were infected with ZIKV at MOI of 2 and incubated for 18 h. The viral supernatant from each time point was collected and submitted to qRT-PCR for quantification of RNA. The results showed low RNA levels when the treatment was performed 0.3, 9.9, or 12 h.p.i. No significant reduction of RNA was observed in the early phases of the replicative cycle (0–1 h.pi), in the release step (after 14 h.p.i), or in the pretreatment condition (−1 h.p.i). In addition, it was analyzed whether the compound Bromocriptine inhibited ZIKV NS2B-NS3 protease in an in vitro system. For this analysis, ZIKV NS2B-NS3 protease containing a fluorescent reporter was previously synthesized, incubated with different concentrations of Bromocriptine for 10 minutes, and then subjected to fluorescence reading. The results showed that Bromocriptine inhibited NS2B-NS3 protease activity in a dose-dependent manner. In addition, in silico analysis showed an intracellular interaction between Bromocriptine and NS2B-NS3 protease. This analysis revealed that Bromocriptine presented high binding affinity with various residues of the NS2B-NS3 complex and could possibly interact with the active site of ZIKV NS2B-NS3 protease and inhibits its activity [39].

The flaviviral NS2B-NS3 protease process the ZIKV polyprotein into seven non-strucutural and three structural proteins, being crucial for ZIKV replication and demonstrating to be an important antiviral target [68]. The NS3-NS2B gene was cloned into a plasmid, and Rosetta2 (DE3) cells were transformed, cultured, and lysed to extract the protease in a study by Lee and coworkers. The authors performed a high-throughput assay testing 70 synthetic compounds though enzyme reactions, measuring fluorescence intensity. The compounds named 1 and 2 strongly inhibited ZIKV protease with half maximal inhibitory concentration (IC_50_) vales of 5.2 µM and 4.1 µM, respectively. Both compounds strongly bound to the protease catalytic site, competitively inhibiting the protein [69]. Other works have also found ZIKV NS2B-NS3 protease inhibitors, such as the peptidomimetic boronic acid [53], dipeptides [70], Aprotinin [54], novobiocin, and lopinavir/ritonavir [52].

### 3.2. In Silico Interaction Analysis and Natural Drugs

Byler and coworkers generated structural models of ZIKV proteins (NS3 helicases, NS5 methyltransferase, RNA-dependent RNA polymerases, and NS2B-NS3 proteases) by homology modeling techniques to analyze in silico the interactions between these viral proteins and molecules derived from plants. A total of 2263 compounds isolated from herbs, including alkaloids, polyphenols, and terpenoids, were screened. For ZIKV NS2B-NS3, only the bis-indole alkaloids flinderole A and flinderole B were in agreement with the Linpinski′s rules (experimental and computational approaches to estimate solubility and permeability in drug discovery and development settings) [71]. For ZIKV NS3 helicase, the isoquinoline alkaloids cassiarin D, 3-omethyldiplacone, exiguaflavanone A, the sesquiterpenoids lactucopicrin, auronekanzonol V, chalcones angusticornin B, balsacone B, and kanzonol Y, and the lignans hibalactone and kaerophyllin showed high binding activity to the ATP site. The polyphenolic compounds cimiphenol, cimiracemate B, and rose marinic acid presented strong docking to NS5 methyl transferase, while ZIKV NS5 RNA-dependent RNA polymerase presented docking ligand properties to the polyphenoic compounds 4,7-digalloylcatechin, prenylatedchalcone, 2,4,4-trihydroxy-3,3-diprenylchalcone, bis-indole alkaloid flinderole, and lignan di-O-demethylisoguaiacin. All polyphenolic compounds showed to be the best ligands, demonstrating high affinity to several protein sites due to the higher potential to the target viral proteins. Monoterpenoids and triterpenoids showed the lowest binding affinity. Other phytochemicals, such as balsacone B, kanzonol V, cinnamoylechinaxanthol, cimiphenol, and rosemarinic acid, isolated from *Populusbalsamifera, Glycyrrhizaglabra, Echinacea root, Cimicifugaracemosa* and *Rosmarinus officinalis*, respectively, showed great docking properties to viral proteins, which highlights the antiviral potential of molecules available in nature [57].

#### 3.2.1. Potential Inhibitors of the Early Stages of Replication

Kuiavanen and coworkers investigated the antiviral activity of the compounds SaliPhe, obatoclax, and gemcitabine in human telomerase reverse transcriptase-immortalized retinal pigment (RPE) cells infected with ZIKV FB-GWUH-2016 strain. These three compounds present anticancer properties and also demonstrated antiviral properties against the Influenza A virus. SaliPhe and obatoclax act to prevent virus entry into cells, while gemcitabine inhibits viral replication [72]. The authors analyzed ZIKV-mediated cellular apoptosis, transcription, signaling, and metabolism. Immunofluorescence assay showed that these compounds, at non-cytotoxic concentrations, inhibited the synthesis of viral proteins 24 h.p.i. The reduction of RNA viral expression and production of infectious viral particles was also confirmed by RT-qPCR or plaque assay 48 h.p.i., respectively. It was also analyzed whether the compounds inhibited ZIKV-mediated cell death. Initially, short treatments were performed with each compound, starting at 0, 2, 4, 6, and 8 h.p.i. The infected cells were maintained in the presence of each compound for 2 h and then supplied with fresh culture medium. A second experiment was performed by adding each compound 0, 2, 4, 6, 8, and 10 h.p.i, but the culture medium containing the compound was not replaced. For both treatments, cell viability was measured 48 h.p.i. The results demonstrated that cellular viability was maintained when the cells were treated up to 4 h.p.i., and gemcitabine protected the cells when added 10 h.p.i without replacing the medium [48]. These compounds also inhibited ZIKV-mediated activation of caspases 3, 7, and 8, responsible for cellular apoptosis. SaliPhe, obatoclax, and gemcitabine were evaluated by their activity on two signaling phosphoproteins which are mainly affected by ZIKV infection, i.e., the transcription factor cAMP response element binding protein (CREB) and the cyclin-dependent kinase inhibitor 1B (p27). For a better understanding of SaliPhe and obatoclax effects on antiviral genes expression, the cells were treated with the compounds at a concentration of 1 µM and infected with ZIKV at MOI = 8,5. After 10 h, the gene expression profiles were analyzed by RNA quantification. Antiviral gene expression was impaired by SaliPhe and obatoclax, but not by gemcitabine. The research results showed that these compounds presented different mechanisms of action, and, therefore, a combined therapy could be a great strategy against ZIKV infection [48].

Epigallocatechin gallate (EGCG) is a natural polyphenol that exists in high abundance in green tea, with antiviral properties described for human immunodeficiency virus (HIV) [73], herpes simplex virus (HSV) [74], influenza virus (FLU) [75], and hepatitis C virus (HCV), consisting in the inhibition of viral entry into cells [76]. The anti-ZIKV potential of the natural compound epigallocatechin gallate (EGCG) was demonstrated in a research conducted by Carneiro and coworkers. They investigated the virucidal potential of EGCG against ZIKV by using Vero cells infected with ZIKV BR or MR766 strains. First, the compound and the virus were simultaneously incubated for 1 h and then added to the cells for 96 h. After incubation, the viral supernatant was titrated by plaque assay. The results demonstrated that EGCG at 200 and 25 μM reduced the foci number of ZIKV BR and MR766 by a minimum of 90% and 85%, respectively. These data also showed that EGCG acts by strongly inhibiting ZIKV entry into Vero cells [44]. Another study showed that EGCG minimally inhibited the infection caused by ZIKV [77]. This could be explained by the difference in the methodology used by the authors. In this work, Raekiansyah and coworkers investigated EGCG effect on ZIKV by infecting the cells in the presence or absence of EGCG for 5 days and by measuring the levels of viral antigens using an enzyme-linked immunosorbent assay (ELISA). Another interesting factor that may have contributed to the divergence of the results may be related to the different types of strain used in this study [77].

After a high-throughput screening against JEV (Japanese Encephalitis Virus), Wang and coworkers found 28 bioactive compounds which inhibited JEV infection by ≥90%. Among them, nine compounds preserved cell viability by more than 80% of. These compounds were tested against ZIKV by pretreating Vero cell for 1 h and then infecting them with ZIKV MOI 0.2 for 1 h. Forty-eight h.p.i. the cell supernatants were submitted to RNA extraction and qRT-PCR. The cells were lysed, and the viral RNA was quantified. Through these assays, the authors found that manidipine, clinipidine, benidipine, primecrolimus, and nelfinavir mesylate were potential antiviral candidates. Manidipine, clinidipine, and benidipinew significantly reduced ZIKV RNA levels into the infected cells. These and the other compounds also decreased ZIKV RNA levels in the supernatants at concentrations ranging from 4 to 10 µM. The authors explained that more studies are needed to elucidate the mechanism of action of these compounds [78].

Using a microscopy assay, Keiko Rausch and coworkers isolated 38 compounds against ZIKV infection. Among those, nanchangmycin, a natural product isolated from *Streptomyces nanchangensis*, presented a strong anti-ZIKVl activity. The authors pretreated U2OS cells with 2 µM of each compound for 1 h, prior to infection with ZIKV (MEX2-81 at a MOI = 100) for 30 minutes. The cells were then fixed and submitted to confocal microscopy, by using an antibody against ZIKV glycoprotein (4G2). Their results showed that the virions remained on the cell surface, and that nanchangmycin inhibited viral entry by blocking clathrin-mediated endocytosis [47].

Arbuckle and coworkers assessed the antiviral activity of EEZH2/1 histone methyltransferases inhibitors against ZIKV. Telomerase reverse transcriptase-immortalized human foreskin fibroblasts (HFF) cells were pretreated with GGSK 126, GS343, UNC1999, astemiziole, and ACV at concentrations ranging from 5 to 100 µM for 5 h and then infected with ZIKV (H/PF/2013; 30,000 FFU/2.2 × 10^4^ cells). Forty h.p.i., the cells were fixed and stained with pan-*Flavivirus* monoclonal antibody E60, and the foci formation was scored by size with Immuno Spot Macronalyzer. The authors conducted a similar assay also with cells treated after ZIKV adsorption. The compound GSK126 strongly inhibited ZIKV infection at concentrations of 15 and 20 µM when the cells were pretreated and also showed antiviral activity in the post-treatment experiment. The authors suggested that the compounds could activate proinflammatory and immune cell recruitment pathways as their mode of action [45].

Cortex Moutan (CM) is the bark of the peony tree root used in Chinese medicine as a drug with hepatoprotective, anti-inflammatory, and antivirals properties. The natural compound Pentagalloylglucose (PPG) is one constituent of CM and has been shown to interfere with the entry of all HCV virus genotypes into human hepatoma cells as well as primary hepatocytes. The antiviral properties of PPG were also tested on ZIKV infectivity. For this, Vero B4 cells were infected with ZIKV Puerto Rico strain and treated with different concentrations of PPG for 48 h. After this time, the supernatants were harvested, and the viral titer in the supernatants was measured by qRT-PCR. The results showed a dose-dependent decrease of the viral titers in the supernatants, with IC_50_ of 4.1 μM. These data showed that the natural compound PPG not only prevents HCV infection but also has anti-ZIKV proprieties, showing the potential of PPG as an antiviral against both infections [49].

Li and Deng tested synthetic 25-Hydroxycholesterol (25HC) as an antiviral against ZIKV. BHK21 cells were treated with 25HC (1–5 μM) and infected with ZIKV (GZ01/2016 strain 200 PFU/well) 12 h later at 4 °C for 1 h. After incubation, the cells were washed with cold PBS to remove the unbound viruses and incubated 1 h at 37 °C. The virus titers were measured by plaque assay. The compound 25HC inhibited ZIKV internalization up to 100% at 5 μM. In previous works, the authors suggested that this compound inhibited viral entry by suppressing the fusion between the virus and the cell membranes for VSV, HIV, and Nipah virus [50].

Curcumin is a natural compound extracted from the *Curcuma longa L.* root that has broad therapeutic properties, such as anti-inflammatory, anti-microbial, and anticancer [79]. The antiviral properties of curcumin were also evaluated against ZIKV. HeLa cells were pretreated with the compound at different concentrations (from 1 nM to 5 µM) for 2 h, and fresh medium containing the compound and ZIKV MOI = 0.1 was added to the cells for 48 h. The viral supernatant was then collected and titrated by plaque assay. The results showed that concentrations equal to or greater than 100 nM significantly decreased the infection by ZIKV in a dose-dependent manner. RNA from the supernatants was also extracted and quantitated by qRT-PCR for the determination of RNA levels, and the data obtained showed that 5 µM of curcumin was able to reduce the viral RNA levels, corroborating the results from the previous assay. To analyze at which stage of the replicative cycle curcumin would be acting, Hela cells were treated with the compound and infected with ZIKV (MOI = 0.1) at different times (before and after infection). In addition, an assay was proposed to verify whether curcumin inhibited the infection by interfering directly with the virus and preventing ZIKV entry into the cells. To this end, the virus and the compound (1, 10, or 100 µM) were incubated for 1 h and then added to the Hela cells for a further 1 h. The cells and the bound virus were collected and lysed, and the RNA was extracted and quantified by qRT-PCR. The results showed a significant a dose-dependent reduction of viral RNA, showing that this could be a possible mechanism of action for curcumin. The same assays were performed with the curcumin analog compounds demethoxycurcumin and bisdemethoxycurcumin, which also presented anti-ZIKV activity, exhibiting similar cell toxicity as unmodified curcumin [43].

#### 3.2.2. Potential Inhibitors of the Late Stages of Replication

In the search for effective antivirals, research centers have screened compounds which previously demonstrated to possess antiviral activity against other pathogens for their therapeutical potential against ZIKV infection [40]. As an example, nucleoside analogues showed to inhibit the viral replication of the herpes simplex viruses 1 and 2 (HSV-1 and HSV-2) [80], cytomegalovirus (CMV) [81], hepatitis B virus (HBV) [82], human immunodeficiency virus (HIV) [83,84,85,86], and hepatitis C virus [87].

Eyer and coworkers investigated the anti-ZIKV efficacy of a panel of 29 nucleoside analogues by using an infected culture system. Vero cells were infected with ZIKV at a MOI of 0.1 in the presence or absence of compounds ranging from 0 to 100 µM. Five days post-infection (d.p.i.), cell viability was determined by a cytotoxicity assay, and the viral titers were determined by the plaque assay. The nucleoside analogues 7-desaza-2′-C-methyladenosine (7-desaza-2′-CMA), 2′-C-methyladenosine (2′-CMA), 2′-C-methylcytidine (2′ CMC), 2′-C-methylguanosine (2′-CMG), and 2′-C-methyluridine (2′-CMU) reduced the cytopathic effect caused by ZIKV infection, demonstrating the anti-ZIKV potential of these compounds [60].

In another work, Vero cells were infected with ZIKV PA259459 strain (MOI 1) for 1 h, washed, and supplied with 35 μM of Nordihydroguairetic Acid (NDGA), tetra-*O*-methyl nordihydroguaiaretic acid (M_4_N), PF-429242, and fosfatin [59]. Twenty-four h.p.i. a plaque assay demonstrated that the four compounds significantly inhibited ZIKV multiplication. It is known that NDGA disturbs the lipid metabolism, on which the *flavivirus* is highly dependent; therefore, this represents one of the mechanisms of action of this compound.

The antiviral activity of 6-methylmercaptopurine riboside (6 MMpr) was evaluated through RNA quantification, viral titration, and plaque reduction assay in Vero and SII-SY5Y neuronal cells by Valério and coworkers [58]. Vero and SII-SY5Y cells monolayers were infected with ZIKV PE243 at MOI 0.1 for 2 h at 37 °C, washed, and additionated with four concentrations of 6 MMpr. The cell supernatants were collected 120 h.p.i. and submitted to qRT-PCR and virus titration. The results showed that 6 MMpr inhibited viral RNA in a dose-dependent manner, and, at 30.3 µM, the riboside decreased the viral RNA up to 99%. In addition, 30.3 and 60.5 µM of 6 MMpr reduced ZIKV titers by 77.24% and 99.45%, respectively. For SII-SY5Y, the cells were submitted to flow cytometry and immunofluorescence 72 h.p.i. The authors concluded that 6 MMpr reduced ZIKV replication by around 70% at 157 µM; additionally, 6 MMpr reduced ZIKV yields by 99.8%. The authors suggested the use of 6 MMpr during viral RNA synthesis induce mutations and viral replication errors [58].

Sofosbuvir (SOF) is a nucleoside inhibitor of HCV NS5B polymerase used in the treatment of patients who have developed a chronic disease [88,89] Mesci and coworkers investigated the antiviral activity of SOF in the infection caused by ZIKV in vitro and in vivo [90]. First, both 2D models (human neural progenitor cells (NPC) and Vero cells) and 3D neurosphere models were infected with ibH 30656 or Brazil-ZKV2015 ZIKV strains and treated with SOF at different concentrations. The results showed that SOF at the IC_50_ of 13.6 μM and 30.9 μM in NPC and Vero cells, respectively, was able to inhibit viral replication and consequently reduce cell death induced by ZIKV infection in both models. Then, the antiviral properties of SOF were tested in vivo in mouse models. NOD/SCID mice (*N* ≥ 6) were infected with 10^8^ PFU of ZIKV (IBH 30656) and treated with 50 mg/kg SOF daily for 10 days. Then, the mice were sacrificed, and blood samples were collected for analysis. Additionally, 2–3-month-old pregnant female SJL were infected with Pa259459 ZIKV (2 × 10^5^ pfu) and treated for 6 days with SOF at 50 mg/kg. On day 7, the mice were sacrificed to collect fetal samples and the mother′s blood. The samples from both assays were processed for serum separation and submitted to plaque assay to determine the viral titer. In addition, viral RNA was extracted from the serum, and qRT-PCR was performed for quantification of the RNA levels. The results of both plaque assay and qRT-PCR demonstrated a reduction in the viral load, supporting the anti-ZIKV activity of SOF [90]. Sacramento and coworkers also tested sofosbuvir in vitro. A yield-reduction assay was performed by infecting Vero cells for 1 h with ZIKV BR at different MOIs and adding different concentrations of sofosbuvir for 24 h. The ZIKV titer in the supernatant was determined by plaque forming. Also, the authors tested if sofosbuvir could inhibit ZIKV RNA polymerase (ZKRP) isolated from BHK-21 infected with ZIKV. For this, a PCR containing ZKRP ribonucleotides, HEPES, MgCl, and 500 ng of ZIKV RNA was performed in the presence or absence of sofosbuvir. The authors concluded that the compound inhibited ZKRP. Also, BHK 21, SH-SY5Y, Huh-7, and Vero cells were infected with ZIKV and treated with different concentrations of sofosbuvir. The compound strongly inhibited ZIKV replication in Huh-7 and SH-5YSY but not in BHK 21 and Vero cells. ZKRP and sofosbuvir interactions were evaluated in silico. The compound and polymerase strongly bound through two amino acid residues which are critical for ribonucleotide incorporation. This was suggested by the authors as one of the mechanisms of action of sofosbuvir [51].

Although the replication of *flavivirus* occurs in the cytoplasm within the endomembrane system, some papers have shown that NS5 proteins can be found in the nucleus, as has been shown for DENV. NS5 enters the nucleus by a viral replication strategy that occurs through the heterodimeric host transport factor importin (IMP) α and IMP β1 [91]. Both compounds ivermectin and *N*-(4-hydroxyphenyl) retinamide (4-HPR) inhibited the viral infection caused by DENV 1–4 by binding to the interaction site of IMP α/β1 and NS5 protein. Because of the similarity between the NS5 of ZIKV and DENV, it was investigated whether ZIKVNS5 protein could be recognized by IMPs, and if 4-HPR inhibits the transport of NS5 to the nucleus, when it interacts with IMP. In order to measure the interactions between these proteins, an AlphaScreen (amplified luminescent proximity homogeneous assay; PerkinElmer, Waltham, MA, USA) assay was performed. The roboticides was measured using 30 µM His6-ZikaRdRp and 10 µM biotinylated IMPα or 10 µM biotinylated IMPβ1, or ivermectin (positive control) and 4-HPR. The results revealed high affinity of Zika RNA-dependent RNA polymerase (RdRp) for both IMPα and IMPβ1 heterodimers showing that, as observed for DENV NS5 protein, ZIKV NS5 protein is imported into the nucleus by interactions with IMPs. The AlphaScreen assay also showed that 4-HPR is a potent inhibitor of the interaction IMP–ZIKV NS5, with IC_50_ = 1μM. Additionally, the antiviral potential of 4-HPR was evaluated. For this, Vero cells were infected with ZIKV at MOI = 0.1 for 2 h and then treated with different concentrations of 4-HPR for 22 h. The supernatant was collected, and the viral RNA was quantified by plaque assay and qRT-PCR. The results showed that 3 μM of the compound significantly inhibited viral infection, suggesting that the mechanism of inhibition is through the ability of 4-HPR to inhibit the interaction between ZIKV NS5 and IMPs [92]. The chemical nucleus of the compound mefloquine (trifluoromethyl) is present in several compounds with already described therapeutic properties against malaria, cancer, and tuberculosis [41].

Barbosa-Lima et al. synthesized and evaluated the antiviral activity of a number of molecules derived from 2,8-*bis* (trifluoromethyl) quinoline (N1-(2,8-*Bis* (trifluoromethyl) quinolin-4-*yl*) ethane-1,2-diamine 3a, *N*1-(2,8-*Bis* (trifluoromethyl) quinolin-4-*yl*) propane-1,3-diamine 3b, *N*1-(2,8-*bis* (trifluoromethyl) quinolin-4-*yl*) decane-1,10-diamine 3c, *N*-butyl-2,8-*bis* (trifluoromethyl) quinolin-4-amine 3d, 2-(2,8-*Bis* (trifluoromethyl) quinolin-4-*yl*) amino) ethanol 4, and *N*-(2-Chloroethyl)-2,8-*bis* (trifluoromethyl) quinolin-4-amine 5) on ZIKV infection in vitro. For antiviral analysis, Vero cells were infected with ZIKV MOI 0.1 for 1 h and then supplied with culture medium containing different concentrations of the compounds. After 24 h of incubation, the viral supernatants were collected, and the viral RNA was extracted and quantified by qRT-PCR. Compounds 3a, 3b, 4, and 5 showed antiviral activity similar to mefloquine. The compounds showed a minimum of 75% reduction of the viral RNA levels, showing to be promising anti-ZIKV molecules [55].

Merimepodib (MMPD) demonstrated to interfere with ZIKV RNA replication by inhibiting IMPDH (inosine-5′-monophosphate dehydrogenase). Tong and coworkers pretreated Huh7 cells with serial dilutions of MMPD, 12 h before infection with ZIKV MR766 at MOI of 0.06. Two days post-infection, the RNA levels were measured from the cell culture supernatants by qRT-PCR. The researchers also assessed cytotoxicity using the (3-(4,5-dimethylthiazol-2-*yl*)-5-(3-carboxymethoxyphenyl)-2-(4-sulfophenyl)-2H-tetrazolium inner salt) (MTS) protocol to determine half maximal effective concentration (EC_50_) and 50% cytotoxic concentration (CC_50_). MMPD inhibited ZIKV RNA replication, with EC_50_ = 0.6 ± 0.2 µM, CC_50_ > 10 µM, and SI > 17, showing to be a potent antiviral against ZIKV infection [56].

## 4. Concluding Remarks

Since the ZIKV infection is currently a serious public health problem, many research centers have directed efforts in the search for a drug with potent anti-ZIKV activity. In this review, we have discussed some approaches that have been performed to this purpose, being most of the considered drugs either pre-approved by FDA or mainly based on molecules previously documented to possess antiviral effects against other diseases, such as Ebola, Giardíase, Malaria, Hepatitis C, Herpes, Hepatitis B, and syndrome of the human immunodeficiency. Some molecules, such as niclosamide, emricasam, ivermectin, MPA, and daptomycin, have shown to be effective on ZIKV infection in vitro by inhibiting viral entry into the cells and/or replication, or by decreasing cell death induced by infection. Natural compounds, synthetic compounds, and nucleoside analogs have been the subject of research in the search for new molecules that inhibit the infection caused by ZIKV, and some studies have demonstrated the therapeutic potential of these different approaches. Other tools, like the use of molecular docking, have optimized the discovery of new molecules that may turn out to be potential antivirals. Efforts to uncover antiviral drugs that treat ZIKV infection continue since the ZIKV outbreak of the 2016. Most of the tests described above were performed *in vitro*, and clinical trials I and II are required to prove the efficacy of these compounds in humans. Currently, few compounds are in phase I trials, such as Sofosbuvir, 7-DMA, and NITD008, and there is still no treatment against ZIKV infection. Hence, further research is clearly needed for the development of effective and safe antivirals that could be used for the control of ZIKV infection with no significant side effects.

## Figures and Tables

**Table 1:** Compounds tested with potential anti-ZIKV activity.

	Compound	Validated Cell/Test	Anti-ZIKV Strategy	Reference
Drug Repurposing	Bromocriptine	Vero cells	Inhibition of NS2B-NS3 protease	[39]
	Emricasan	Gliobastoma SBN-19, hNPCS and human astrocytes cells	Reduces cellular apoptosis by inhibition of caspase-3 activities	[40]
	Ivermectin, Daptomycin, Mycophenolic acid (MPA), Sertraline, Pyrimethamine, Cyclosporine A, Azathioprine, Mefloquine	HuH 7 cells	Unknow	[41]
	Nicosamide, PHA-690509	Gliobastoma SBN-19, hNPCS and human astrocytes cells	Inhibit viral replication	[40]
	Chloroquine	Vero, hNSC and hBMEC cells	Inhibits early stages of replicative cycle	[42]
	Bromocriptine	Vero cells	Inhibition of NS2B-NS3 protease	[39]
Potential Inhibitors of the early and late stages of replication	Curcumin	HeLa cells	ZIKV entry and virucidal effect	[43]
	Epigalocatequina galato	Vero cells	Inhibits the viral entry	[44]
	GSK126	Telomerase reverse transcriptase-immortalized HFF cells	Strongly inhibited ZIKV infection in pre-treated cells	[45]
	Heparin	human neural progenitor cells (hNPCs)	Inhibits caspase 3 activity mediated by ZIKV infection.	[46]
	Nanchangmycin	U2OS cells	Blocks viral entry blocking clathrin-mediated endocytosis	[47]
	Obatoclax	Human telomerase reverse transcriptase-immortalized retinal pigment (RPE)	Inhibits endocytic uptake of ZIKV and viral protein synthesisPrevents caspase 8, 3 and 7 activationProtects the phosphorylation status of p27 phosphoprotein	[48]
	Pentagalloylglucose (PPG)	Vero B4	ZIKV entry	[49]
	SaliPhe	human telomerase reverse transcriptase-immortalized retinal pigment (RPE)	Inhibits endocytic uptake of ZIKV and protein viral synthesisPrevents caspase 8, 3 and 7 activation	[48]
	25-Hydroxycholesterol (25 HC)	BHKK-21	Inhibits ZIKV internalization	[50]
	Sofosbuvir	HNPCs, Huh-7, SH-5YSY, Vero cells. Neurosphere, in silico	Nucleoside inhibitor; binds too amino acid residues critical for ribonucleotide incorporation; interacts strongly with ZIKV RNA polymerase	[51]
	Novobioctin, lopinavir-ritonavir	Vero/Huh-7/in silico	Inhibition of NS2B-NS3 protease	[52]
	Peptidomimetic boronic acid	Huh-7/*in silico*	Inhibition of NS2B-NS3 protease	[53]
	Aprotinin	*In silico*	Inhibition of NS2B-NS3 protease	[54]
	*N*-(4-hydroxyphenyl) retinamide (4-HPR)	Vero cells	Inhibition of viral replication probably interacting with NS5 protein	[55]
	Merimepodib (MMPD)	Huh7	Inhibits ZIKV RNA replication inhibiting IMPDH (inosine-5′-monophosphate dehydrogenase)	[56]
	Gemcitabine	Human telomerase reverse transcriptase-immortalized retinal pigment (RPE)	Interferes with transcription of viral RNAInhibits viral protein synthesisPrevents caspase 8, 3 and 7 activationChanges the phosphorylation status of the CREB phosphoprotein affected by ZIKV infection	[48]
	Cimiphenol, Cimiracemate B, Rosemarinic acid	*In silico*	high affinity with NS5 methyl transferase	[57]
	6-methylmercaptopurine riboside (6 MMpr)	Vero and SII—SY5Y neuronal cells	6 MMpr used during viral RNA synthesis reducing the viral infectivity caused by mutations and viral replication errors	[58]
	4,7-digalloylcatechin, Prenylated chalcone, 2,4,4-trihydroxy-3,3-diprenylchalcone,Bis-indole alkaloid flinderoleLignan di-*O*-demethyl isoguaiacin	*In silico*	High affinity with NS5 RNA-dependent RNA polymerase	[57]
	Bis-indole alkaloids flinderole A and flinderole B	*In silico*	High affinity with NS2B-NS3 protease	[57]
	Cimiphenol, Cimiracemate B, Rosemarinic acid	*In silico*	High affinity with NS5 methyl transferase	[57]
	Isoquinoline alkaloid cassiarin D3-Omethyldiplacone, Exiguaflavanone A, Sesqui terpenoid lactucopicrinAurone kanzonol V, Chalconesvangusticornin BBalsacone Bkanzonol Ylignans hibalactone kaerophyllin	*In silico*	Inhibition of NS3 helicase ATP site	[57]
	Nordihydroguairetic Acid	Vero	Disturbs the lipid metabolism	[59]
	Nucleoside analogues: 7-desaza-2′-C-methyladenosine (7-desaza-2′-CMA)2′-C-methyladenosine (2′-CMA)2′-C-methylcytidine (2′ CMC)2′-C-methylguanosine (2′-CMG)2′-C-methyluridine (2′-CMU)	Vero, Human neuroblastoma (UKF-NB-4 and porcine kidney (PS) cells	Reduce cytopathic effect, cell death and inhibit the viral replication	[60]
